# Cognitive Control and Individual Differences in Economic Ultimatum Decision-Making

**DOI:** 10.1371/journal.pone.0027107

**Published:** 2011-11-09

**Authors:** Wim De Neys, Nikolay Novitskiy, Leen Geeraerts, Jennifer Ramautar, Johan Wagemans

**Affiliations:** 1 Centre National de la Recherche Scientifique (CNRS), University of Toulouse, Toulouse, France; 2 Lab Experimental Psychology, University of Leuven, Leuven, Belgium; 3 Department of Economics and Applied Economics, University of Leuven, Leuven, Belgium; 4 Netherlands Institute for Neuroscience, Amsterdam, The Netherlands; Kyushu University, Japan

## Abstract

Much publicity has been given to the fact that people's economic decisions often deviate from the rational predictions of standard economic models. In the classic ultimatum game, for example, most people turn down financial gains by rejecting unequal monetary splits. The present study points to neglected individual differences in this debate. After participants played the ultimatum game we tested for individual differences in cognitive control capacity of the most and least economic responders. The key finding was that people who were higher in cognitive control, as measured by behavioral (Go/No-Go performance) and neural (No-Go N2 amplitude) markers, did tend to behave more in line with the standard models and showed increased acceptance of unequal splits. Hence, the cognitively highest scoring decision-makers were more likely to maximize their monetary payoffs and adhere to the standard economic predictions. Findings question popular claims with respect to the rejection of standard economic models and the irrationality of human economic decision-making.

## Introduction

In recent years much publicity has been given to the fact that people's economic decisions often deviate from the rational predictions of traditional economic models [1]–[3]. One of the prime showpieces of people's apparently irrational economic behavior is provided by a simple game known as the Ultimatum Game [4]. In the game two players have to split a sum of money. One player (the proposer) makes an offer as to how the money should be split between the two. The other player (the responder) can either accept or reject this offer. If the responder accepts, the deal goes ahead as planned. However, if the responder rejects the offer, then neither player receives anything. Both players are fully aware of the rules of the game and once the decision is made, the game is over.

Standard economic models prescribe that the optimal solution is for the proposer to offer as little as possible, and for the responder to accept this small amount on the rational grounds that earning something is better than earning nothing. In economic game theory this solution is known as the Nash Equilibrium (after the Nobel prize winning economist J. F. Nash). However, numerous experimental studies over the last two decades have indicated that people's actual behavior does not resemble the Nash Equilibrium predictions: Proposers most commonly offer an even split (i.e., the money is split 50∶50) and responders typically reject an uneven split in which they are only offered a small amount [1].

Psychological research indicated that people's decision to reject is driven by strong emotional motives [3], [5]–[9]. Uneven splits and small offers are perceived as unfair and provoke an angry reaction. This strong emotional reaction leads people to sacrifice considerable financial gain in order to punish their partner for the slight. Hence, contrary to the century old characterization of the human decision-maker as motivated by rational deliberation, human economic decisions seemed to be primarily driven by emotional impulses.

While these findings have helped economists to start taking psychological and emotional factors into account they also led to a questioning of the standard economic models and the rationality of human decision-makers (e.g., [1], [3]). This questioning has been amplified by ultimatum research in special populations that showed that, for example, chimpanzees or very young children do tend to accept unfair splits [10], [11]. Ironically, such findings seemed to suggest that behaving in line with the standard economic models is characterized by less cognitive sophistication. Clearly, this tends to further undermine the value of the standard economic models [1]. As one popular science website put it, the fact that the “homo economicus” envisaged by the standard models turns out to be a monkey is not making economist or the human race look good [12].

In the present paper we point to a basic but somewhat neglected issue in this debate. Studies of economic decision-making in the general population have typically focused on explaining aggregate behavior with little interest paid to individual differences [13]. For example, in the ultimatum game, researchers' attention has been primarily captured by the fact that *most* healthy adults typically reject uneven splits. However, although the majority of responders rejects uneven splits, there is always a small minority who does accept them and behaves in line with the standard economic model. Clearly, a key question is what characterizes this minority. In the present study we explore the possibility that the tendency to accept unfair offers is mediated by cognitive control abilities. We hypothesize that people who have a superior cognitive capacity to override impulsive behavior will better manage to control the emotional impulse to reject uneven splits. Consequently, contrary to what might seem to be suggested by primate or developmental research, we predict that in the general human population, individuals with the highest control abilities should be more likely to adhere to the rational Nash Equilibrium solution.

Our basic hypothesis was inspired by individual differences studies on logical and probabilistic reasoning (e.g., [14]–[16]). These studies indicated that although most people are typically biased when they engage in logical or probabilistic reasoning, participants highest in cognitive control capacity do manage to reason in line with standard logical or probabilistic models. More specifically, it has been shown that a high control ability allows high control reasoners to override erroneous but highly salient intuitively cued responses that bias their less fortunate counterparts (e.g., [16]–[18]). Clearly, in and by itself, the fact that one is more likely to behave in line with a standard logical or probabilistic model in a reasoning task, does not imply that one will also be more likely to behave in line with standard economic models during economic decision-making (but see [19]). However, the fact that cognitive control capacity has been shown to help control impulsive intuitive responses during reasoning lends some credence to the idea that it might also help to counter the cued emotional impulses in the ultimatum game.

More specific support for our hypothesis comes from neuroimaging work with the ultimatum game (e.g., [3], [20]). Sanfey and colleagues, for example, showed that unfair offers mainly elicit activity in brain areas that are related to emotional (anterior insula) and cognitive control processing (lateral prefrontal cortex). The critical finding was that for accepted unfair offers the lateral prefrontal activation was stronger than the insula activation, whereas the reverse was true for rejected unfair offers [3]. The lateral prefrontal cortex has been linked to cognitive control processes such as overriding impulsive responses [21]. Hence, although the imaging findings do not allow us to draw strong individual differences conclusions they at least suggest that increased cognitive control processing is associated with more economic responses (e.g., [20]). In the present study we tested our claim directly by examining the link between individuals' ultimatum game performance and behavioral and neurological correlates of their cognitive control capacity. We also validated the functionality of cognitive control resources for economic decision-making by examining ultimatum game performance under cognitive load.

In the study we first invited a large number of participants for an initial screening session in which they played the Ultimatum Game in the role of responder. Based on the screening we invited a group of the most and least economical responders (i.e., participants who most and least accepted unfair offers) for a follow-up study in which they were presented with a Go/No-No task while electroencephalography (EEG) was recorded. The Go/No-Go task is a classic task that is widely used to measure people's basic cognitive control abilities (e.g., [22], [23]). In the task participants must quickly respond to a frequently presented Go stimulus such that the ‘Go’ response becomes habitual. However, on a small proportion of trials, a No-Go stimulus appears, signaling that one's habitual response should be withheld. Hence, a No-Go stimulus conflicts with the prepotent Go response tendency. People's accuracy on the No-Go trials is an excellent marker of their basic ability to control impulsive responding. Consequently, if we are right that individuals who accept unfair offers are characterized by higher cognitive control abilities, we predict that at the behavioral level, the most economical responders will excel in the Go/No-Go task and outperform the least economical responders.

The EEG recording allowed us to test for a possible neurological marker of the differential control capacities of the least and most economical thinkers. Correctly solved No-Go trials on which participants manage to withhold the dominant ‘Go’ response give rise to a specific event-related potential (ERP) component referred to as the No-Go N2. The No-Go N2 is a sharp negative voltage deflection in the EEG that typically peaks about 200 ms after the stimulus onset. The No-Go N2 is believed to reflect cognitive control activity associated with successful monitoring or overriding of the prepotent Go response [23].

Available evidence suggests that the few times that people with less developed cognitive control abilities do manage to withhold the Go response, the N2 amplitude is larger than for people with high control abilities (e.g., [24]–[28] but see also [29], [30]). Some have interpreted this larger No-Go N2 amplitude as reflecting the fact that people who have fewer cognitive control resources will need a much higher activation of the neural control structures for the response inhibition to be successful [27], [28]. Others, such as Nieuwenhuis and colleagues [23], argued that the No-Go N2 reflects a conflict related reaction to the No-Go stimulus as a cue to change one's prepotent behavioral response. Under this interpretation the No-Go N2 amplitude may reflect the degree of conflict that is required to change one's habitual response rather than the response override per se. People with high control ability will be more accurate on No-Go trials precisely because they are more responsive to conflict and will change their habitual response at the slightest sign of it. People lower in control ability will need much more conflict-related activation in order to achieve this habitual response change. Consequently, the few times that individuals low in cognitive control manage to change their prepotent Go response and solve a No-Go trial correctly, this will be associated with high levels of conflict-related activation and larger No-Go N2 amplitude.

In sum, although there is some debate regarding the precise interpretation of the No-Go N2, it is well established that individual differences in cognitive control ability affect the No-Go N2 amplitude. Consequently, if economical responders in the ultimatum game are indeed characterized by high cognitive control abilities, we should not only observe a different behavioral No-Go performance in the two groups but also a differential, presumably smaller, No-Go N2 component for the most economic responders.

## Methods

### Ethics statement

All experiments in this study were conducted in accordance with the Declaration of Helsinki and approved by the local ethics committee of the University of Leuven. Written informed consent was obtained from all participants.

### Ultimatum Screening

#### Participants

A total of 403 psychology undergraduates participated in return for course credit. Ten participants were randomly selected and received additional payment based on their earnings in the ultimatum game. Participants provided written informed consent and the study was approved by the local ethics committee of the University of Leuven.

#### Material

**Figure 1:**
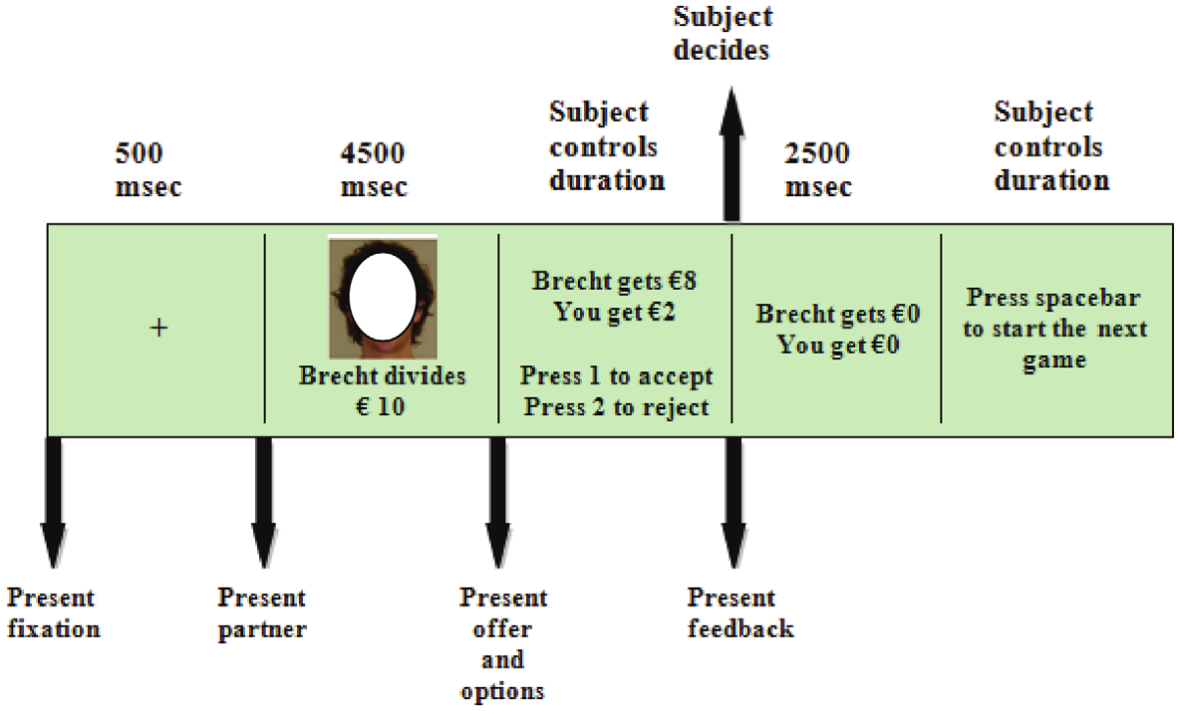
Timeline for a single ultimatum game round. Note that the player picture is anonymized for publication.

*Ultimatum Game*. Our ultimatum game procedure was based on the work of van't Wout and Sanfey (e.g., [3], [5]). Participants played a total of 10 one-shot games with 10 different proposers. Participants always played the role of the responder. Participants were presented with a picture of their proposer, after which the proposal was presented and participants could accept or reject the offer (see Figure 1). Participants were clearly instructed that they would play a single round of the game with each proposer and that the proposers were not informed about the participants' decisions in previous rounds. Each round involved splitting €10. The offers adhered to a predetermined algorithm. Half of the presented offers were control trials in which the proposer offered to split the money evenly (€5: €5). The other half were the critical trials in which the participants were presented with an unequal split in which the proposer wanted to keep the larger part (two offers of €9: €1, two offers of €8: €2, and one offer of €7: €3). We will refer to the control and critical trials as fair and unfair trials, respectively. The different offers were presented in random order. Participants were informed that 10 randomly selected participants would receive the total amount of money they made in the game.

*Cognitive Reflection Test (CRT).* After participants finished playing the ultimatum game they completed a number of unrelated tasks and were presented with the Cognitive Reflection Test (CRT, see [15]). The CRT is a very short, 3-item questionnaire designed to measure people's ability to refrain from impulsive responding in a reasoning context. The test shows good correlations with standard cognitive ability tests and quantitative SAT scores. The brief test was translated in Dutch (see [31]) and included as a raw proxy of people's cognitive control capacities. The measure allowed us to have a first, explorative look at the association between an individual's behavioral control ability and ultimatum game performance.

### Go/No-Go EEG Study

#### Participants

After the ultimatum screening nine of the least and nine of the most economical responders (i.e., screening study participants whose unfair trial acceptance rate scored in the bottom and top quartile, respectively) were recruited for the main Go/No-Go EEG study. Participants were paid €25 for their participation. Participants provided written informed consent and the study was approved by the local ethics committee of the University of Leuven.

#### Material

*Go/No-Go task*. The Go/No-Go task was based on the procedure introduced by Nieuwenhuis and Amodio (e.g., [22], [23]). On each trial, either the letter “M” or “W” was presented in the center of a computer screen. Approximately half of the participants in each group were instructed to make a “Go” response (mouse button press) when they saw “M” but to make no response when they saw “W”; the remaining participants completed a version in which “W” was the Go stimulus and “M” the No-Go stimulus. Each trial began with a fixation point, presented for 500 ms. The target then appeared for 100 ms, followed by a blank screen. Participants were instructed to respond within 500 ms of target onset. A warning message appeared on the screen for 1 s after responses that exceeded this deadline and after erroneous responses. The inter trial interval was 1 s.

The task consisted of 600 trials: 80% Go trials and 20% No-Go trials. The high frequency of Go trials induced a prepotent “Go” response, enhancing the difficulty of successfully overriding a response on the critical No-Go trials. Participants received a short 2-min break after every 150 trials. As one reviewer remarked, one might note that the Go/No-Go task involves the override of an experimentally acquired tendency whereas the ultimatum game involves the override of an alleged “natural” tendency. In theory this could affect the predictive power of the Go/No-Go task. Nevertheless, prior research already showed that the Go/No-Go task is a good predictor of the override efficiency across a wide-range of domains (e.g., [25], [27], [28]).

### Procedure

#### EEG recording

Participants were fitted with a Quickcap, and EEG was collected from 128 equidistantly positioned scalp sites using Ag/AgCl electrodes. The active reference electrode was placed on the vertex between electrodes Cz and Cpz. A ground electrode was placed on the forehead close to AFz. Vertical and horizontal electrooculogram (EOG) was collected to permit the reduction of the artifact due to eye movements. Impedances were below 5 kΩ at each scalp site. EEG was recorded through a 0.15–30 Hz bandpass filter and digitized at 1000 Hz using a SynAmps2 amplifier. Data were re-referenced to the average earlobe. Offline, we used a computerized algorithm [32] to remove eye-blink artifacts. EEG epochs with voltage exceeding +/- 200 µV were rejected as reflecting additional artefact.

#### ERP N2 processing

Our quantification of the N2 was based on the work of Amodio and colleagues [22]. A 1000 ms epoch of EEG signal, beginning 200 ms prior to stimulus onset, was selected for each artifact-free trial. Baseline correction procedures subtracted the average voltage during the 200 ms interval before stimulus onset within each epoch from the entire epoch. Epochs associated with correct responses on Go and No-Go trials were averaged within their respective trial types. The N2 was scored as the peak negative deflection occurring between 200 and 400 ms, relative to target onset, at the vertex site (Cz), where it is typically maximal. The critical No-Go N2 component refers to the average N2 amplitude associated with correct “No-Go” responses. For control purposes we also calculated the average N2 amplitude, scored according to the same criteria, associated with correct “Go” responses. We will refer to this component as the Go N2.

### Ultimatum Load Study

If our Go/No-Go EEG study were to show that more economic ultimatum responders are indeed characterized by better cognitive control capacity, this does not yet establish that the better economic performance is driven by cognitive control resources per se. That is, it cannot be excluded that other factors account for the association. We already noted, for example, that chimpanzees and young children also tend to accept unfair offers (e.g., [10], [11]). This behavior has been attributed to the fact that these populations show no or, in the case of children, a far less intense emotional reaction to an unequal split. The same point has been made with respect to the remarkably high unfair offer acceptance rate of autistic patients and certain tribes living in isolated cultures, for example [1]. Hence, in sharp contrast with the typical western adult human, accepting an unfair offer does not require overriding any prepotent emotional response in these special groups. Clearly, given this override redundancy, the high acceptance rate in these groups is not surprising. However, the issue does point to a possible alternative explanation for our findings. One might argue that, just like monkeys, people higher in cognitive control capacity are simply less emotional and do not need to override an emotional response to accept an unfair offer. Hence, the higher cognitive control capacities of people who behave more economically might be an epiphenomenal coincidence and play no functional role in their economic decision-making.

To address this issue we asked a group of participants with superior cognitive control capacities to play the ultimatum game while their control resources were burdened with a demanding secondary task. If people's superior cognitive control capacities are critical to override emotional impulses, cognitive load will reduce the efficiency of the override and hamper performance. However, if people with a superior control capacity are simply less emotional and their cognitive control capacity is not functional for their economic behavior, their ultimatum performance should not be affected by an experimental reduction of the available cognitive resources.

### Participants

We selected 36 participants for the ultimatum load study based on an initial screening in which 200 undergraduates were presented with a Go/No-Go task. The Go/No-Go task was similar as in the EEG study except that participants played only 150 trials. Participants who scored in the top quartile were invited for participation in the ultimatum game study. Participants received course credit in return for participation in the screening and ultimatum study. In addition, five ultimatum participants were randomly selected and received payment based on their earnings in the ultimatum game. All participants provided written informed consent and the study was approved by the local ethics committee of the University of Leuven.

### Material

**Figure 2:**
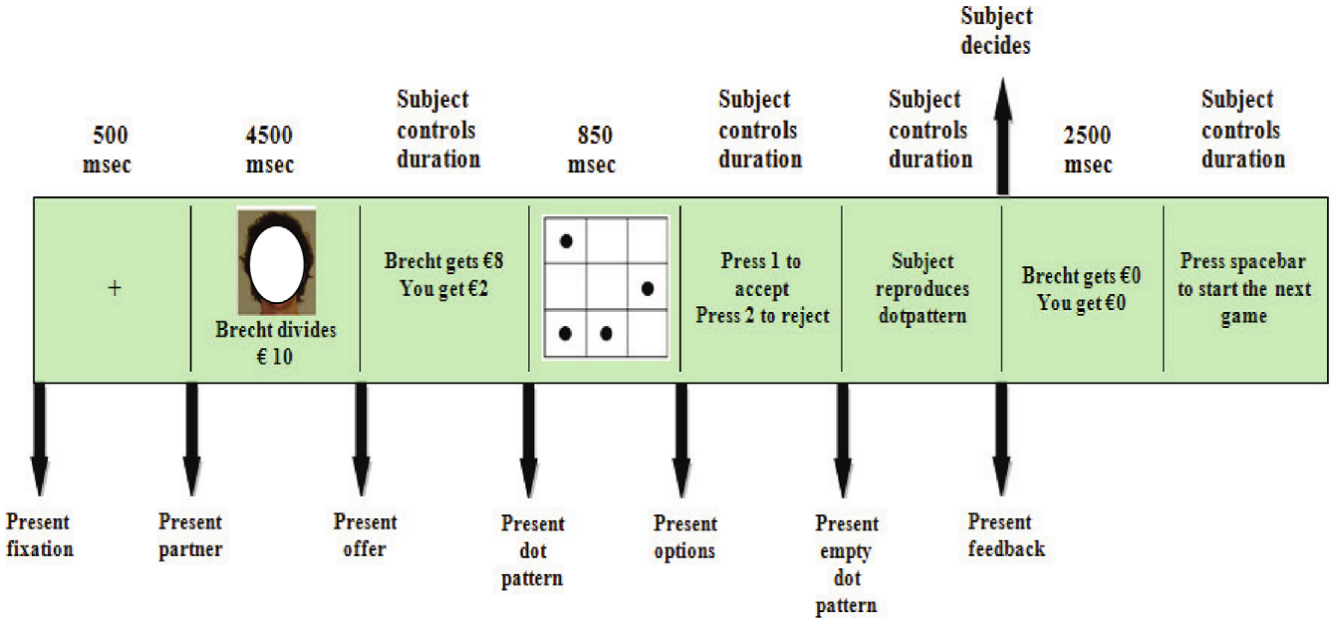
Timeline for a single ultimatum game round under secondary task load. Note that the player picture is anonymized for publication.

Half of the selected participants were randomly asked to play the ultimatum game under dual task load. The other half played without additional load. A control analysis established that the cognitive control capacity, as measured by the No-Go accuracy, did not differ in the load and no load group, F(1, 34)<1. The ultimatum game was similar as in our EEG screening study. The dual task procedure was based on the work of De Neys [17]. Participants in the load group were presented with a to-be-memorized dot pattern before the offer was presented. After participants had entered their response an empty grid appeared and participants were asked to reproduce the dot pattern (see Figure 2). The dot memorization task has been shown to efficiently tap executive control resources [33].

## Results

### Ultimatum Screening

The ultimatum game behavior of our screening sample replicated the typical results in previous studies. Overall, people tended to reject an unequal split in which the proposer wanted to keep the largest part. Average acceptance rate for the unfair trials was only 20% (*SE* = 1.3). The larger the part the proposer wanted to keep, the less the unfair offer was accepted (7∶3 split = 45%, *SE* = 2.1; 8∶2 split = 17%, *SE* = 1.3; 9∶1 split = 11%, *SE* = 1.3, F(2, 804) = 156.25, p<.00001, 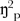 = .28). Equal splits, however, were typically accepted. In contrast with the unfair trials, the acceptance rate reached 98% (*SE* = .3) on the fair control trials, F(1, 402) = 3502.9, p<.00001, 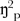 = .9.

The average CRT score in the sample was 1.12 (out of 3, *SE* = .05). As we expected, there was a significant correlation between people's acceptance rate on the unfair ultimatum trials and their CRT scores, r = .27, p<.00001. Hence, people who tended to accept the critical unfair offers did show higher CRT scores. Obviously, acceptance rates of the almost perfectly accepted fair offers did not depend on CRT performance, r = −.01, p = .7740. This establishes that participants with low CRT scores do not simply show a general tendency to reject offers. On the fair trials, where the money is split evenly, people with lower CRT scores do accept the offer. As one would expect, it is only when the money is split unequally and accepting requires overriding a negative emotional impulse that individual differences in cognitive control (as measured by the CRT at least) will matter. Non-parametric correlation tests confirmed the findings (Spearman rank-order correlation CRT-unfair trials acceptance = .19, p = .0002; Spearman rank-order correlation CRT-fair trials acceptance  = .−.04, p = .3864). The interested reader can find an illustration of these trends in Figure S1.

Two months after the initial screening we invited a group of the least (i.e., participants who never accepted an unfair offer) and most economical responders (i.e., participants who accepted unfair offers more than once) to participate in the main EEG study. These cutoff values corresponded to the top and bottom quartile of the acceptance rates on the unfair trials. All participants were invited by email to participate. Time-constraints forced us to restrict the number of recruited participants for the EEG study to the first nine participants from each group who responded.

### Go/No-Go EEG Study

#### Behavioral findings

**Figure 3:**
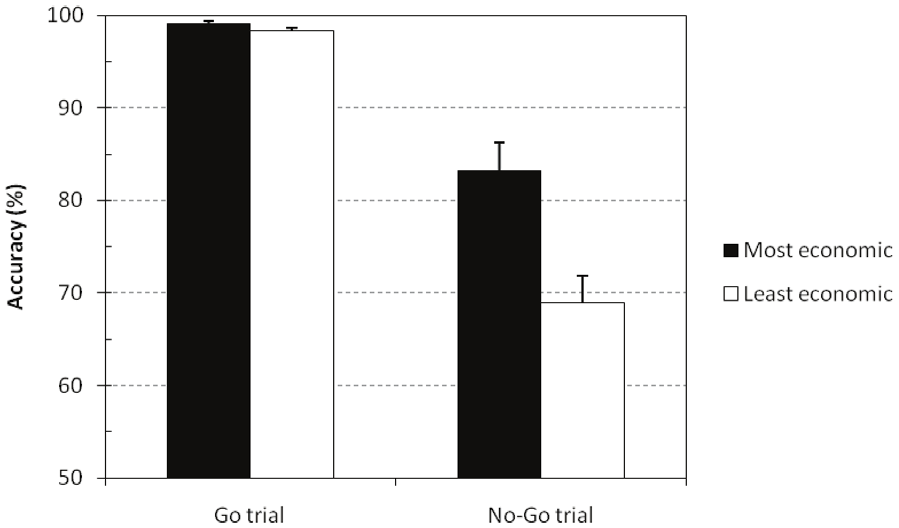
Go/No-Go task accuracy. Error bars are standard errors.

The average accuracy on the Go and No-Go trials of participants in our group of least and most economic ultimatum game responders was entered in a 2 (Go/No-Go trial)×2 (economic group) mixed model ANOVA. Figure 3 shows the results. There was a main effect of the trial type, F(1, 16) = 119.48, p<.00001, 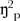 = .88, and economic group factor, F(1, 16) = 11.50, p = .0037, 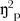 = .42. The two factors also interacted, F(1, 16) = 10.60, p = .0049, 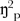 = .40. Planned contrasts showed that, as expected, the most economic responders outperformed the least economic group on the critical No-Go trials where correct responding required controlling the impulsive Go response, F(1, 16) = 11.14, p = .0042, 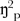 = .75. As Figure 3 shows, both groups performed equally well on the Go trials where overriding the impulsive response was not required, F(1, 16) = 3.58, p = .0766.

#### Neurological findings

**Figure 4:**
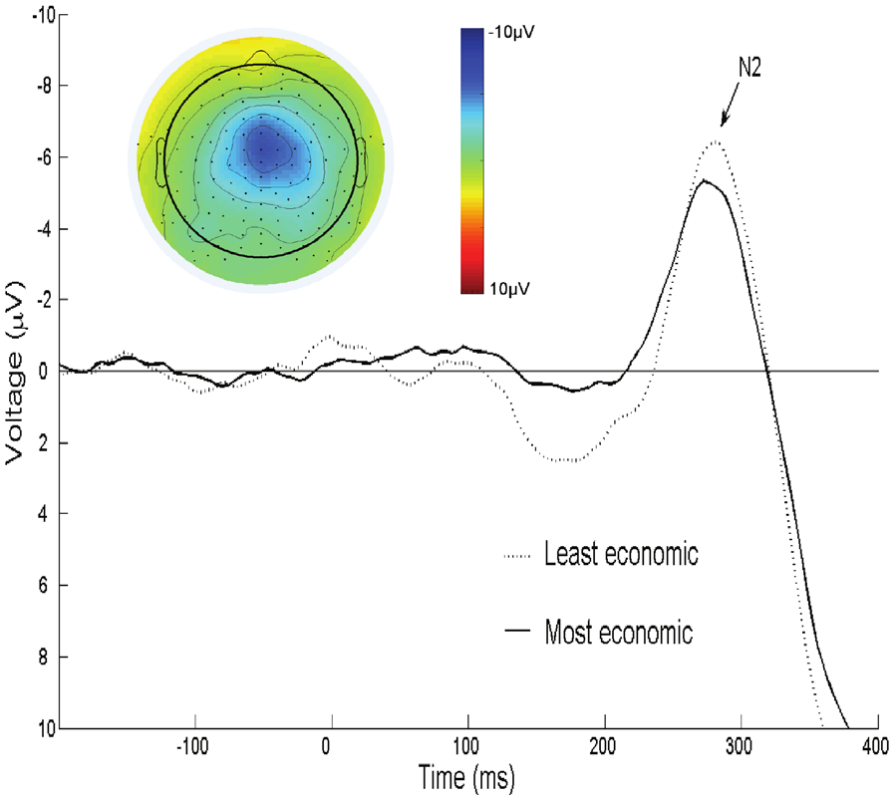
ERP waveforms. Waveforms corresponding to correct No-Go responses, with the waveform for correct Go responses subtracted, for the least and most economic ultimatum game responders are shown (stimulus presented at 0 ms; N2 peaked at 278 ms at Cz). The inset shows the voltage map of the scalp distribution of the resulting N2.

As expected, our ERP data indicated that the average No-Go N2 amplitude differed in the group of most and least economic thinkers, F(1, 16) = 6.85, p = .0187, 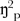 = .30. As Figure 4 shows, whenever the least economic thinkers did manage to solve No-Go trials correctly this was accompanied by a more pronounced No-Go N2 amplitude (i.e., a more negative deflection). A control analysis established that the least economic thinkers did not simply show a general tendency towards more negative ERP deflections. The Go N2 (i.e., our control ERP associated with correctly solved Go responses, scored to correspond to the No-Go N2) did not differ for the most and least economic responders group, F(1, 16)<1. Hence, in line with the behavioral findings, the N2 only differed when correct responding required overriding an impulsive response.

**Figure 5:**
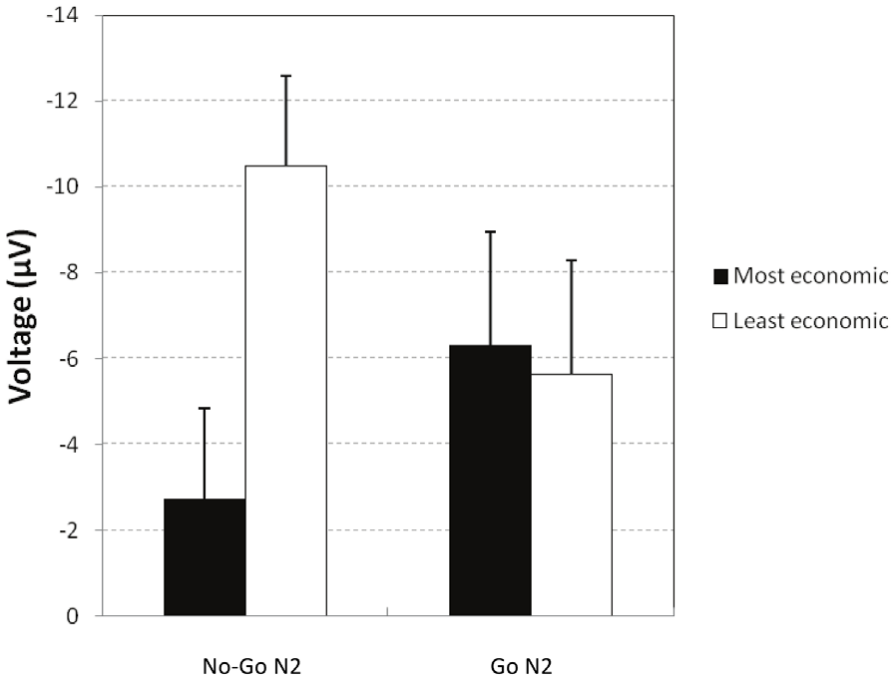
Average peak No-Go and peak Go N2 amplitude. Errors bars are standard errors.

For completeness, we also entered the No-Go N2 and control Go N2 data in a mixed model ANOVA with Trial type (No-Go or Go) as within-subjects factor and Economic Group (least or most economic) as between-subjects factor. The main effects of Trial Type, F(1, 16)<1, and Economic Group, F(1, 16) = 1.59, were not significant, but as expected the two factors interacted, F(1, 16) = 4.49, p = .0499, 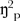 = .22. As indicated above, the N2 only differed significantly between the groups on the critical No-Go trials, F(1, 16) = 6.85, p = .0187, 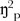 = .30. This pattern is illustrated in Figure 5.

### Ultimatum Load Study

**Figure 6:**
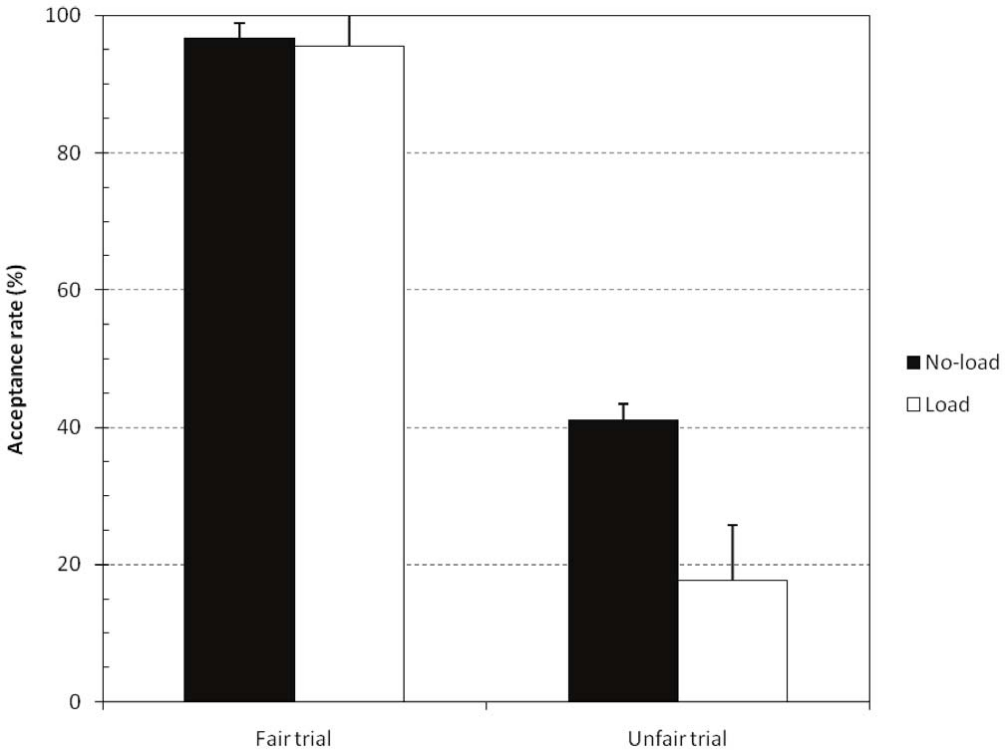
Impact of cognitive load on fair and unfair offer acceptance. Error bars are standard errors.

An initial control analysis indicated that the load task was properly performed. On average 94% (i.e., 3.76 out of 4 dots, SE = .12) of the dots were correctly memorized. Next, the acceptance rates on fair and unfair trials of participants in the load and no-load group were entered in a 2 (fair or unfair trial)×2 (load or no-load group) mixed model ANOVA. Figure 6 shows the results. There was a main effect of the trial type, F(1, 34) = 129.66, p<.00001, 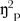 = .79, and load factor, F(1, 34) = 4.40, p = .0433, 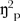 = .12. As Figure 6 indicates, the two factors also tended to interact, F(1, 34) = 3.60, p = .0662, 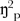 = .10. Planned contrast showed that the cognitive load decreased the acceptance rates on the critical unfair trials, F(1, 34) = 4.31, p<.0456, 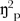 = .11. However, the load did not affect performance on the fair trials, F(1, 34)<1. This pattern establishes that the decreased acceptance rate on the unfair trials cannot be attributed to a dual task confound. It is not the case that cognitive load simply results in a general tendency to reject offers. As expected, cognitive capacity only matters on the unfair trials where the unequal split is expected to cue an impulsive rejection response.

## Discussion

In the present study we pointed to neglected individual differences in economic decision making. In line with previous psychological studies we observed that contrary to the predictions of standard economic models, the vast majority of participants turned down monetary gains and rejected unequal splits when playing the ultimatum game. However, the key finding is that people who are higher in cognitive control, as measured both by behavioral (Go/No-Go performance) and neural markers (No-Go N2 amplitude), do tend to behave more in line with the standard economic models and are more likely to accept unequal splits. Our cognitive load study established that this increased acceptance behavior is indeed mediated by the cognitive control resources. Hence, the cognitively highest-scoring decision-makers' economic behavior does tend to more closely resemble the game theoretic Nash Equilibrium predictions. The net consequence is that these cognitively highest-scoring players will be the ones who end up with the highest monetary gains.

We noted that the observed deviations of people's behavior from the standard economical predictions have resulted in a questioning of the economic models and the rationality of human decision-makers [1], [3]. Since the standard models describe how a perfectly rational being that maximizes its payoffs should behave, people's failure to behave in line with these prescriptions can be interpreted as pointing to the irrationality of the human species. At the same time, one can also argue that the models' prediction failures point to a need to improve and revise the models. The present individual differences findings indicate that both these claims need to be qualified. Although there might be a lot of people whose behavior conflicts with rational predictions, some people do tend to behave more rationally. Our study indicates that these decision-makers are typically those individuals highest in cognitive control ability. This should help to counter strong claims with respect to the irrationality of the human economic decision-makers. In addition, the individual differences also qualify the claim to revise the standard models. Clearly, for some people, specifically those highest in cognitive control abilities, economic decisions are much more in line with the predictions of the standard models. Any proposed revision of the standard models will need to take this individual variance into account.

We mentioned that neuroimaging studies have indicated that accepting unfair offers is associated with increased lateral prefrontal activation (e.g., [3], [20]). As we pointed out, in line with the present findings, this lateral prefrontal region is believed to mediate cognitive control processes such as overriding impulsive responses [21]. However, with respect to the precise neural basis of the rejection override in the ultimatum game it is interesting to consider the recent findings of Knoch and colleagues (e.g., [34]-[36]). Using both repetitive transcranial magnetic stimulation (rTMS) and transcranial direct current stimulation (tDCS), Knoch and colleagues showed that deactivation of the (dorsal) lateral prefrontal cortex resulted in an increased acceptance of unfair offers (see also [37]). As Knoch and colleagues argued, this either indicates that it is the *rejection* of unfair answers that requires control resources or that the lateral prefrontal cortex is involved in the mediation of the emotional response to unfairness. Van't Wout and colleagues [37] and Tabibnia and colleagues [20] already argued for this latter possibility and suggested that the dorsolateral prefrontal cortex (DLPFC, i.e., the part of the lateral prefrontal cortex that was deactivated in the Knoch [35], [36] and van't Wout [37] studies) is specifically involved in emotional goal maintenance. Hence, when rTMS or tDCS deactivate this dorsolateral area, subjects will no longer experience a willingness to reject unfair offers (because the “rejection” goal is no longer maintained). Bluntly put, rTMS or tDCS over the DLPFC would turn participants in virtual monkeys who no longer feel an emotional trigger to reject and consequently have little trouble in accepting the unfair offer. Note that under this interpretation it is the more ventral part of the lateral prefrontal cortex (VLPFC) that is believed to be responsible for the actual response override and mediation of cognitive control related processing during the ultimatum game. Consistent with this view, Tabibnia and colleagues [20] already observed that a higher acceptance rate of unfair offers was more strongly associated with VLPFC than DLPFC activation. Interestingly, in light of the present findings this suggests that individual differences in cognitive control capacity might also be specifically reflected in a differential VLPFC (rather than DLPFC) recruitment during the ultimatum game. Obviously, the present study was not designed to address this localization question and the hypothesis will need to be validated in future studies.

With respect to the implications of our study it should be clear that we do not argue that cognitive control capacity is the *only* factor affecting ultimatum game performance. The goal of our study was to point to the role of individual differences in cognitive control capacity in economic decision making. We demonstrated that people who are higher in cognitive control tend to behave more in line with traditional models and are more likely to accept unfair offers. However, as we stated, it has been shown that monkeys, young children, or people with certain disorders also accept unfair offers (e.g., [1], [10], [11]). Hence, it is evident that not everybody who accepts an unfair offer will necessarily have a superior cognitive capacity. Likewise, not everybody with a superior cognitive capacity will always accept unfair offers. This point is underlined by our CRT analysis which indicates that the relation between cognitive capacity and the unfair offer acceptance is far from perfect. As we clarified, the idea is that cognitive control capacity matters because it is needed to override the emotional impulse to reject an uneven split. Obviously, any factor that affects the intensity of the emotional response will by definition also affect one's ultimatum behavior. Hence, if for one or the other reason the emotional response is not generated or less intense, unfair offers will also be accepted irrespective of one's control capacities. On the other hand, in the presence of factors that increase ones emotional reactivity to unfair offers (e.g., possible psychopathic personality traits, see [38]) even high control capacities might not suffice to counter the rejection response. In general, previous ultimatum game research has already pointed to the mediating role of such factors as the role of social concerns and the perceived intentions of the proposer (e.g., [36], [39], [40]). For example, some people might be instinctively driven to accept unfair offers to preserve their reputation and preventing them to be perceived as someone who is so poor or so attracted by money to accept little sums [36]. Cleary, our study does not argue against the role of these factors. To the extent that they can modulate or bypass the emotional rejection response they will directly affect the mediating role of cognitive control capacity. What we tried to highlight, however, is that the overall relationship between cognitive control capacity and unfair ultimatum offer acceptance is positive and that our load findings establish that cognitive control directly facilitates the acceptance of unfair offers. This presents an interesting addition to the widely publicized ultimatum game studies with special populations and clarifies that behaving in line with traditional economic standards is not necessarily characterized by less cognitive sophistication.

## Supporting Information

Figure S1 Figures illustrating the association between ultimatum game performance and Cognitive Reflection Test (CRT) scores. Panel A (fair trials) and panel B (unfair trials) show frequency scatterplots. Panel C shows the average acceptance of unfair and fair trials as a function of CRT score.
(TIF)
