# Supplementary figures and images for: Cognitive Control and Individual Differences in Economic Ultimatum Decision-Making
Source: PLoS One. 2011 Nov 9;6(11):e27107. doi: 10.1371/journal.pone.0027107 (PMC3212542; doi:10.1371/journal.pone.0027107)

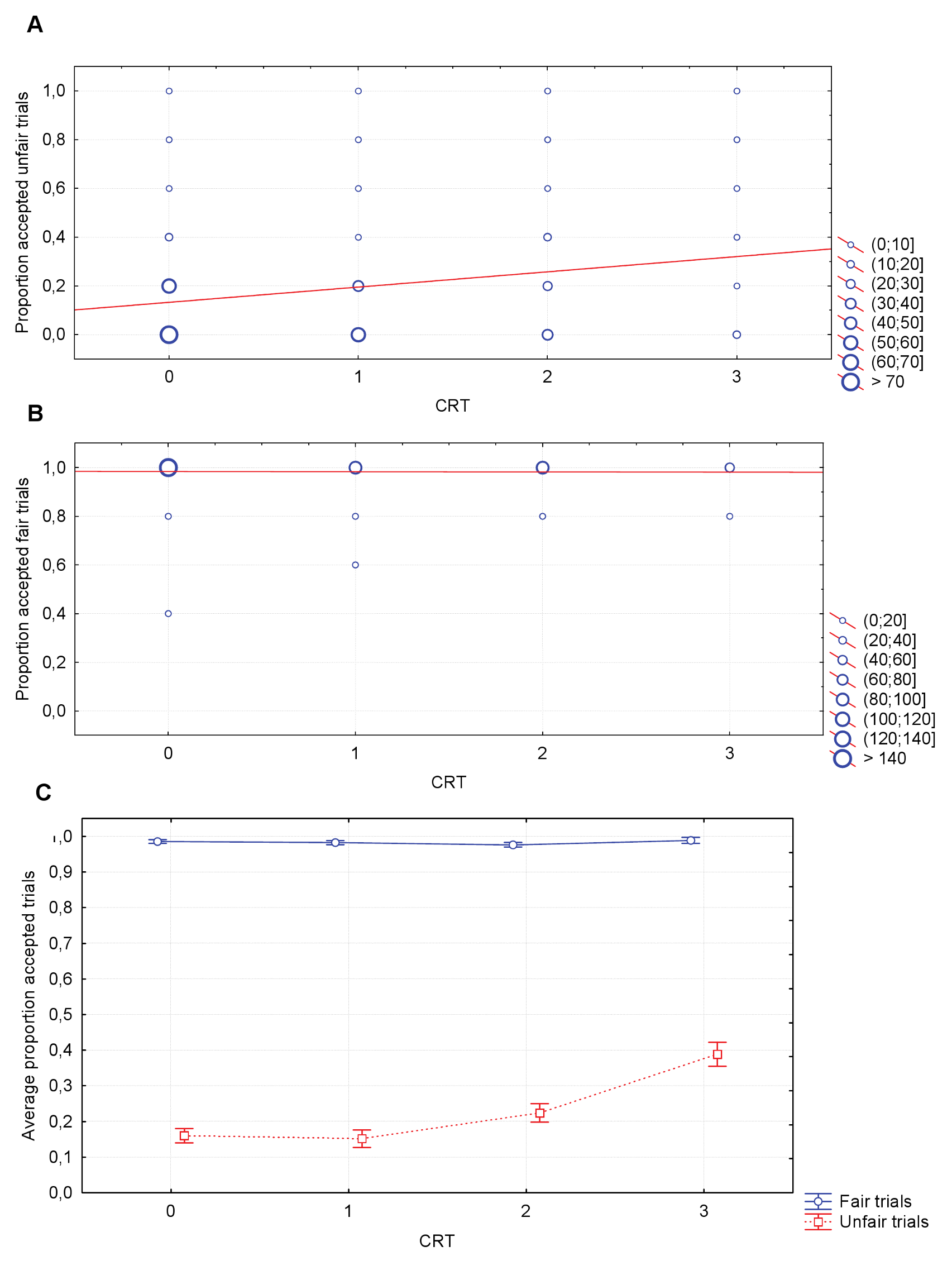

Supplement: Figure S1 — Figures illustrating the association between ultimatum game performance and Cognitive Reflection Test (CRT) scores. Panel A (fair trials) and panel B (unfair trials) show frequency scatterplots. Panel C shows the average acceptance of unfair and fair trials as a function of CRT score. (TIF) [file pone.0027107.s001.tif]
